# Skin cancer special issue in Skin Health and Disease

**DOI:** 10.1002/ski2.224

**Published:** 2023-03-10

**Authors:** Oriol Yélamos, Shamir Geller, Selin Tokez

**Affiliations:** ^1^ Dermatology Department Hospital de la Santa Creu i Sant Pau Universitat Autònoma de Barcelona Barcelona Spain; ^2^ Institut d’Investigació Biomèdica Sant Pau (IIB SANT PAU) Barcelona Spain; ^3^ Department of Medicine Dermatology Service Memorial Sloan Kettering Cancer Center New York New York USA; ^4^ Department of Dermatology Tel Aviv Sourasky Medical Center Sackler Faculty of Medicine Tel Aviv University Tel Aviv Israel; ^5^ Department of Dermatology Erasmus Medical Center Cancer Institute University Medical Center Rotterdam Rotterdam The Netherlands

1

Skin cancer is a major health issue since it is the most common cancer worldwide and its incidence is rising year after year.^1^, ^2^, ^3^, ^4^ Although most skin cancers are non‐lethal, they lead to significant morbidity and high treatment costs. Therefore, primary prevention strategies, such as sun prevention, or secondary prevention strategies, including early detection using dermoscopy or reflectance confocal microscopy, are crucial.^1^, ^5^, ^6^, ^7^

In addition, certain skin cancers, including melanoma, squamous cell carcinoma or Merkel cell carcinoma can be very aggressive and result in significant mortality.^8^, ^9^ This has been especially significant during the COVID‐19 pandemic where mortality rates related to skin cancers have increased, potentially due to delayed detection resulting in more advanced tumours.^10^ Hence, it is crucial to ensure skin cancer screening and surveillance in order to improve detection and treatment.

In this special issue of *Skin Health and Disease* we aimed to highlight the complexity of skin cancers by publishing a wide array of manuscripts covering different aspects related to skin cancers, ranging from primary prevention strategies related to sun education,^11^ genetics,^12^ and epidemiology^13^, ^14^, ^15^, ^16^ to surgical^17^, ^18^ and non‐surgical^19^, ^20^, ^21^ treatments. From these papers, we would like to highlight some hot topics such as the paper from Borg et al^17^ evaluating the impact of the COVID‐19 pandemic in the surgical management of skin cancers on the head and neck area in elderly patients. Another relevant paper evaluates the impact of 3D total photography, a novel system developed to screen populations at high risk for skin cancer, in terms of skin cancers excised and the histopathology outcomes.^18^ We have left some room for non‐invasive skin imaging such as reflectance confocal microscopy which has been used by Han et al^20^ to monitor the treatment of lentiginous melanoma with imiquimod.

Additionally, we did not forget to include studies describing rare tumours or rare associations such as cutaneous metastatic endometrial carcinoma,^22^ a comprehensive review of malignant chondroid syringoma,^14^ or a systematic review about the association between melanoma and genital lichen sclerosus.^13^ We believe that a better knowledge of infrequent skin cancers is of utmost importance in order to improve the understanding and management of such tumours.

We also aimed to advance understanding of how skin cancer presents in different ethnicities with a manuscript comparing the melanoma outcomes in black versus white patients.^16^ Although most skin cancers are rare in non‐white populations, most studies to date have focussed on white populations resulting in a knowledge bias that is essential to address.

Due to its high incidence and large heterogeneity in terms of clinical presentation and management, skin cancer continues to be a trending topic. In the last years, this has resulted in an improved understanding of the genetic pathways of skin tumours, the development of efficacious medical treatments such as immunotherapy and targeted therapies, and the advent of novel treatment strategies such as neoadjuvant approaches. We hope this issue will reflect this exciting time in skin cancer research and encourage more research to improve patient outcomes.

## CONFLICT OF INTEREST STATEMENT

Oriol Yélamos is an Associate Editor of SHD. Oriol Yélamos has received consultancy honoraria from Almirall, Bioderma, Isispharma Kiowa Kirin and Leo Pharma, and speakers' honoraria from Almirall, BMS, Isispharma, Jannsen‐Cilag, La Roche Posay, Leo Pharma, MSD and Pierre Fabre. SG is an Associate Editor of SHD. Shamir Geller has received speakers' honoraria from Takeda and Rafa. Selin Tokez is an Associate Editor of SHD.

## AUTHOR CONTRIBUTIONS

**Oriol Yélamos**: Writing – original draft (lead); Writing – review & editing (lead). **Shamir Geller**: Writing – original draft (supporting); Writing – review & editing (supporting). **Selin Tokez**: Writing – original draft (supporting); Writing – review & editing (supporting).

## FUNDING INFORMATION

This editorial received no specific grant from any funding agency in the public, commercial, or not‐for‐profit sectors.

## ETHICS STATEMENT

Not applicable.

## Data Availability

Data sharing is not applicable to this article as no new data were created or analysed in this study.
